# Electroceuticals for Regeneration of Long Nerve Gap Using Biodegradable Conductive Conduits and Implantable Wireless Stimulator

**DOI:** 10.1002/advs.202302632

**Published:** 2023-06-20

**Authors:** Jio Kim, Jooik Jeon, Ju‐Yong Lee, Badamgarav Khoroldulam, Sung‐Geun Choi, Jae‐Young Bae, Jung Keun Hyun, Seung‐Kyun Kang

**Affiliations:** ^1^ Department of Materials Science and Engineering Seoul National University Seoul 08826 Republic of Korea; ^2^ Department of Nanobiomedical Science and BK21 NBM Global Research Center for Regenerative Medicine Dankook University Cheonan 31116 Republic of Korea; ^3^ Department of Rehabilitation Medicine College of Medicine Dankook University Cheonan 31116 Republic of Korea; ^4^ Institute of Tissue Regeneration Engineering (ITREN) Dankook University Cheonan 31116 Republic of Korea; ^5^ Research Institute of Advanced Materials (RIAM) Seoul National University Seoul 08826 Republic of Korea; ^6^ Nano Systems Institute SOFT Foundry Seoul National University Seoul 08826 Republic of korea

**Keywords:** biodegradable conductive conduit, electrotherapy, long nerve defects, peripheral nerve regeneration, wireless electrical stimulation

## Abstract

Regeneration of over 10 mm long peripheral nerve defects remains a challenge due to the failure of regeneration by prolonged axotomy and denervation occurring in long‐term recovery. Recent studies reveal that conductive conduits and electrical stimulation accelerate the regeneration of long nerve defects. In this study, an electroceutical platform combining a fully biodegradable conductive nerve conduit and a wireless electrical stimulator is proposed to maximize the therapeutic effect on nerve regeneration. Fully biodegradable nerve conduit fabricated using molybdenum (Mo) microparticles and polycaprolactone (PCL) can eliminate the unwanted effects of non‐degradable implants, which occupy nerve paths and need to be removed through surgery increasing the risk of complications. The electrical and mechanical properties of Mo/PCL conduits are optimized by controlling the amounts of Mo and tetraglycol lubricant. The dissolution behavior and electrical conductivity of biodegradable nerve conduits in the biomimetic solutions are also evaluated. In in vivo experiments, the integrated strategy of a conductive Mo/PCL conduit with controlled therapeutic electrical stimulation shows accelerated axon regeneration for long sciatic nerve defects in rats compared to the use of the Mo/PCL conduit without stimulation and has a significant therapeutic effect based on the results obtained from the functional recovery test.

## Graphical abstract

An electroceutical platform is designed to accelerate nerve regeneration and improve functional recovery by combining fully biodegradable conductive nerve conduit fabricated using rolled thin films of Mo/PCL composites with a wireless electrical stimulator. The motor and sensory function recovery is up to 4 weeks faster with electrical stimulation than without stimulation.

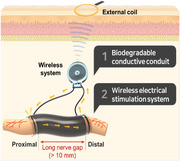

## 1 Introduction

Peripheral nerve injury causes the impairment of sensory and motor functions, muscle atrophy, and neurogenic pain, with most cases of severe peripheral damage recovering incompletely.^[^ ^1^, ^2^ ^]^ A completely transected peripheral nerve can be repaired using end‐to‐end neurorrhaphy, but long nerve gaps over 10 mm require connecting materials.^[^ ^3^ ^]^ Autologous nerve grafting is the current gold standard in the clinical field, but it is limited in supply and can cause donor site morbidity and sensory loss.^[^ ^4^, ^5^ ^]^ Cellular or acellular allografts from cadavers are also used in place of autograft; however, problems such as immune response have not been completely resolved, and its axonal regeneration ability is less effective.^[^ ^3^ ^]^

Alternatively, artificial nerve conduits (ANCs) have been suggested for nerve regeneration due to advantages such as reducing neuroma, scars, and lateral germination, showing no donor site morbidity, and unlimited source of supply.^[^ ^5^, ^6^, ^7^, ^8^ ^]^ An ANC connects the distal and proximal ends of the injured nerve and provides structural support for neurite regeneration and protection from the surrounding tissue such as fibrous connective tissue.^[^ ^5^, ^6^, ^7^, ^8^ ^]^ Ultimately, ANCs guide the axons from the proximal stump in the injured nerve to grow toward the distal target.^[^ ^5^, ^6^, ^7^, ^8^ ^]^ Biodegradable and biocompatible polymers, such as poly(lactic acid) (PLA), poly(glycolic acid) (PGA), poly(lactic‐co‐glycolic acid) (PLGA), and polycaprolactone (PCL), are widely used as ANC materials because of their appropriate mechanical properties.^[^ ^8^, ^9^ ^]^ The use of these polymers as ANCs prevents chronic foreign body reactions and the formation of fibrotic tissue that causes nerve compression, and there is no risk of removal surgery.^[^ ^5^ ^]^ In addition, the introduction of porous/groove and multichannel structures as mechanical cues,^[^ ^10^, ^11^, ^12^ ^]^ nerve growth factors,^[^ ^13^, ^14^ ^]^ and conductive substrates^[^ ^15^, ^16^ ^]^ as biochemical cues, and support cells as biological cues^[^ ^14^, ^17^ ^]^ to ANCs further enhances the regenerative effect.

Especially, the use of conductive substrates can increase the proliferation and differentiation of nerve cells and support cellular activity because of the electro‐active nature of the nerve tissue.^[^ ^18^, ^19^, ^20^ ^]^ Previously reported biodegradable conductive nerve conduits (CNCs) for long nerve regeneration were fabricated by blending conductive polymers, primarily polypyrrole (PPy), or graphene with biodegradable polymers. Examples of polymer blends include PPy/Chitosan (CS), PPy/poly(d, l‐lactic acid) (PDLLA), and PPy/silk fibroin (SF).^[^ ^16^, ^21^, ^22^ ^]^ CNCs consisting of carbon or graphene‐based materials in composite form with biodegradable polymers include carbon nanotube‐interfaced phosphate glass microfibers/poly(L/D‐lactic acid) (CNT‐PGFs/PLDLA), graphene oxide/PCL (GO/PCL), reduced graphene oxide‐coated *Antheraea pernyi* silk fibroin/poly(L‐lactic acid‐co‐caprolactone) (rGO‐coated *Ap*F/PLCL), and rGO/gelatin‐methacrylate (GelMA).^[^ ^1^, ^23^, ^24^, ^25^ ^]^ In these CNCs, conductive polymers and graphene are not degradable; thus, the degradation of the matrix polymer results in conductive additive residues. Hence, they cannot be categorized as biodegradable CNCs. Owing to their incomplete degradation in the body, removal surgery is required, and the biocompatibility issue remains unresolved. CNCs can achieve high conductivity and complete biodegradability using biodegradable metal and biodegradable polymer. Biodegradable metals include magnesium (Mg), iron (Fe), zinc (Zn), and Mo. These materials have been investigated for applications in the field of transient electronics, medical stents, and biomedical engineering, to name a few.^[^ ^26^, ^27^, ^28^, ^29^ ^]^ Mo has been reported as biocompatible in previous studies and showed neither inflammation issues nor accumulation in the liver or kidneys after degradation.^[^ ^30^ ^]^ Studies on the conductivity range of substrates in nerve regeneration are limited, but the conductivity of previously studied CNCs is very low.

Recently, electrical stimulation is highlighted to prompt the regeneration of nerves and recovery of their functions because it 1) promotes axonal growth in the right direction, 2) accelerates initial nerve regeneration and thus, shortens the overall regeneration period, and 3) promotes reinnervation and functional recovery.^[^ ^31^ ^]^ Al‐Majed et al. reported that a low‐frequency electrical stimulation increased axonal regeneration and preferential motor reinnervation in a rat femoral nerve transection and repair model.^[^ ^31^, ^32^, ^33^ ^]^ Since then, many studies have revealed that electrical stimulation is effective for nerve regeneration.^[^ ^34^, ^35^, ^36^, ^37^, ^38^, ^39^, ^40^ ^]^

Only a limited number of studies have been conducted that combine both CNCs and electrical stimulation to promote nerve regeneration and functional recovery in long nerve gaps.^[^ ^21^, ^22^, ^41^, ^42^, ^43^ ^]^ Previous studies primarily rely on the percutaneous wire‐type stimulation through the skin, which poses a significant risk of infection during long‐term treatment.^[^ ^21^, ^22^, ^42^ ^]^ In particular, such a system is unsuitable for clinical applications because it necessitates surgery to insert a wire under the human skin and remove it whenever stimulation is required. Battery systems integrated with CNCs even in biodegradable form do not offer control over stimulation parameters such as pulse duration, stimulation pulse frequency, the number of stimulation days, or intensity.^[^ ^41^, ^43^ ^]^ Control of stimulation conditions and parameters is particularly crucial in long nerve gaps, as they are yet to be optimized. Electrical stimulation conditions for nerve regeneration must consider factors such as pulse duration, stimulation pulse frequency, and the number of stimulation days. Previous studies have demonstrated that several days of stimulation is effective for nerve regeneration. Based on prior research, short‐term (1 h) daily stimulation is more effective for axonal regeneration than continuous stimulation.^[^ ^44^ ^]^ When stimulation is performed for 1 h a day for 1, 3, and 6 days, nerve regeneration and reinnervation effects improve with the number of days.^[^ ^29^ ^]^

In this study, we propose an electroceutical platform integrated with a fully biodegradable CNC and controllable wireless electrical stimulator. We fabricated a composite type of nerve conduit with enhanced conductivity by blending biodegradable metal Mo particles with a biodegradable PCL. The conduit was designed to show good biocompatibility and gradually degrade within a desired period to maintain a certain level of conductivity during nerve regeneration. Moreover, electrical stimulation can be controlled wirelessly at the desired period and time by introducing an inductive coupling‐based wireless stimulator, and continuous wireless electrical stimulation was applied to maximize the nerve regeneration effect through controlled therapeutic stimulation. The effect of CNC and electrical stimulation was demonstrated through controlled therapeutic stimulation by applying the electroceutical to a long nerve defect of the rat sciatic nerve. In vivo experiments revealed accelerated axon regeneration for long nerve defects compared to the use of the Mo/PCL conduit without stimulation.

## 2 Results and Discussion

### 2.1 Electroceutical Platform Consisted of Biodegradable Conductive Nerve Conduit and Wireless Electrical Stimulator

**Figure 1:**
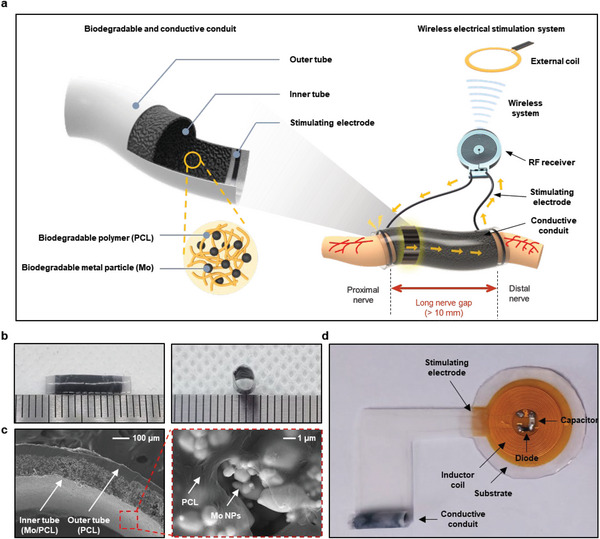
Electroceuticals consisted of a biodegradable conductive conduit and a wireless electrical stimulator for nonpharmacological peripheral nerve regeneration of long nerve gaps. a) Schematic illustration of a biodegradable and conductive conduit (left) and its application to long nerve gaps with the integration of a wireless stimulator (right). Biodegradable and conductive conduit is composed of an outer tube made of PCL and an inner tube made of a Mo/PCL composite. The stimulating electrode made of stainless steel connected to the RF receiver is attached to the outer tube of the conduit, and the RF receiver and conduit are connected to form a system, which converts the power signal transmitted from the external coil into stimulation through the RF receiver and allows electrical stimulation to be transmitted to the nerves through the stimulating electrode. b) Photograph of a biodegradable CNC with ≈14 mm long outer tube, ≈10 mm long inner tube, and inner diameter of ≈2 mm. (tick mark: 1 mm) c) Cross‐sectional SEM image of biodegradable and CNC (left), SEM image of the inner tube (Mo/PCL composite) (right). d) Photograph of the electroceutical using biodegradable and conductive conduit and wireless electrical stimulator employed in this study.

**Figure** **1**a shows a schematic illustration of the regenerative treatment of a long nerve defect using an electroceutical platform consisting of a biodegradable conductive conduit and a wireless electrical stimulator. The fully biodegradable CNC is composed of an ≈50 µm thick outer tube made of PCL and an ≈100 µm thick inner tube made of a Mo/PCL composite with Mo microparticles (MPs) of ≈1.33 ± 0.38 µm. The SEM image of Mo MPs is shown in Figure S1 (Supporting Information). A biodegradable CNC is designed to physically and electrically connect the proximal and distal stump separated by a 10 mm long nerve gap. In addition, wireless electrical stimulator scavenging externally generated RF electromagnetic waves provides controlled stimulation from the proximal to the distal stump through the CNC. Figure S2 (Supporting Information) shows the structure of a wireless electrical stimulator consisting of an inductor coil, a capacitor, and a diode. The capacitor, diode, and stimulating electrode are soldered on the substrate containing the inductor coil and after soldering, all the components are encapsulated with Ecoflex 0030. After the encapsulation, the final thickness and size of our stimulator are 1.2 and 2 mm, respectively. Figure S3 (Supporting Information) shows the rectifier circuit of the wireless electrical stimulator and the circuit of the external coil.^[^ ^29^ ^]^ A stimulating electrode (stainless steel, 50 µm in diameter) interconnects the wireless stimulator with the outer tube of the conduit. The actual lengths of the outer and inner tubes in the biodegradable and conductive conduit are ≈14 and ≈10 mm, respectively (Figure 1b). The inner tube diameter of ≈2 mm is similar to that of the sciatic nerve in rats. The cross‐sectional scanning electron microscope (SEM) image of the biodegradable CNC (left) and inner tube SEM image of Mo/PCL composite (right) in Figure 1c demonstrate uniformly dispersed Mo MPs in the PCL matrix. Figure 1d shows a photograph of a fully integrated wireless electroceutical with biodegradable CNC used in this study.

### 2.2 Electrical and Mechanical, and Dissolution Properties of Mo/PCL Composites

**Figure 2:**
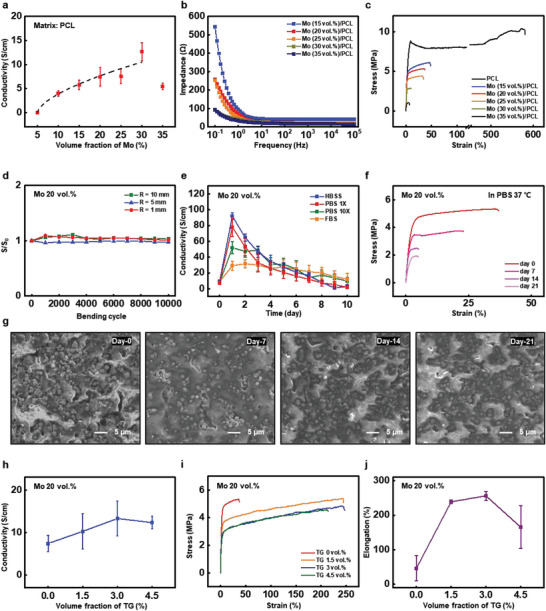
Characterization of electrical and mechanical properties of biodegradable and conductive Mo/PCL composites without and with the addition of tetraglycol (TG) as a lubricant. a) Electrical conductivity of Mo/PCL composites depending on the volume fraction of Mo MPs (≈1.33 ±  0.38 µm) in the PCL matrix. The symbols represent the experimental data, and the dotted lines is the fit based on the percolation theory. b) Electrochemical impedance spectroscopy of Mo/PCL composites consisting of various volume fractions of Mo MPs. c) Uniaxial tensile stress‐strain curves of Mo/PCL composites depending on the volume fractions of Mo MPs. (black, 0 vol.%; blue, 15 vol.%; red, 20 vol.%; orange, 25 vol.%; green, 30 vol.%; and navy, 35 vol.%) d) Change in electrical conductivity of Mo/PCL composites (20 vol.% of Mo MPs) after 10000 cycles of bending at three different bending radii (R = 1, 5, and 10 mm). e) Time‐dependent electrical conductivity of Mo/PCL composites (20 vol.% of Mo MPs) immersed into various simulated body fluid solutions at 37 °C. (blue, Hank's balanced salt solution (HBSS); red, phosphate‐buffered saline (PBS) of 1X concentration; green, PBS of 10X concentration; and orange, fetal bovine serum (FBS)) f) Stress‐strain curves of Mo/PCL composites (20 vol.% of Mo MPs) at various stages of immersion into 1X PBS at 37 °C. g) SEM images showing surface morphologies of Mo/PCL composites (20 vol.% of Mo MPs) at different stages of the immersion into 1X PBS at 37 °C. h) Electrical conductivity, i) uniaxial tensile stress‐strain curves, and j) Elongation of Mo/PCL composites (20 vol.% of Mo MPs) depending on the added amount of TG.

As shown in **Figure** **2**a, the electrical conductivity of Mo/PCL composites as a function of Mo MPs volume fraction increases with the increasing concentration of conductive Mo MPs, according to percolation theory expressed in Equation 1:^[^ ^45^, ^46^ ^]^

(1) $$\sigma =\sigma_{0}{\left(\varphi -\varphi_{c}\right)}^{t}$$

where *σ*, *φ*, *φ*_*c*_, *σ*_0_, and *t* are the conductivity of the composite, the volume fraction, the critical volume fraction of the conductive phase (percolation threshold), the scaling factor, and the critical exponent, respectively.^[^ ^45^, ^46^ ^]^ Here, the critical volume fraction and the critical exponent were 5 vol.% and ≈0.6, respectively. The Mo/PCL composite with the Mo MPs volume fraction (vol.%) of ≈30% showed the highest conductivity of ≈12.66 ± 1.88 S cm^−1^. When the volume fraction reaches ≈35%, the conductivity decreases because the dispersion of metallic fillers becomes non‐uniform due to excess of Mo MPs (Figure S4, Supporting Information).^[^ ^47^ ^]^ Figure 2b presents the results obtained from the impedance measurements of Mo/PCL composites with various Mo MPs volume fractions as a function of frequencies (10^−1^–10^5^ Hz). Evidently, the impedance of Mo/PCL composites decreases as the concentration of Mo MPs increases for all frequency ranges.

Figure 2c shows uniaxial tensile stress–strain curves of Mo/PCL composites with various Mo MPs volume fractions, revealing that both elongation and failure stress decrease with the increasing number of Mo MPs. Similar changes in mechanical properties were previously observed for composites consisting of inorganic fillers and biodegradable polymer matrices, such as PLGA, polybutylene adipate terephthalate (PBAT), and PCL.^[^ ^48^, ^49^, ^50^, ^51^, ^52^ ^]^ When particles are added to the polymer matrix, elongation and tensile strength decrease due to the immobilization of the polymer chains by the particles.^[^ ^52^ ^]^ Stress concentration occurs locally at the interface between the matrix and the particles, resulting in accelerated crack generation and failure at lower stress. As shown in Figure 2a, the conductivities of 15, 20, 25, and 30 vol.% Mo content are 5.8 ± 0.97, 7.4 ± 1.9, 7.5 ± 1.5, and 12.66 ± 1.88 S cm^−1^, respectively, and the conductivity is maximum at 30 vol.% of Mo. In the results presented in Figure 2c, when the Mo content is over 30 vol.%, the elongation decreases significantly and the composite becomes brittle, making it unsuitable for CNC. For the Mo MPs content between 15, 20, and 25 vol.%, there is a minor difference in the elongation at break values, which are 47, 35, and 30, respectively. However, at 15 vol.% Mo, the impedance value is relatively high at low frequencies, making the 20 vol.% Mo more suitable for CNC. The conductivity range of PPy/CS, PPy/PDLLA, and PPy/SF, which are CNCs composed of a conducting polymer, is 1 × 10^−3^–1.57 × 10^−2^ S cm^−1^.^[^ ^16^, ^21^, ^22^ ^]^ And the conductivity range of CNT‐PGFs/PLDLA, GO/PCL, rGO‐coated *Ap*F/PLCL), and rGO/gelatin‐methacrylate (GelMA), which are CNCs composed of carbon or graphene‐based material, is 1 × 10^−6^−1.03 × 10^−2^ S cm^−1^.^[^ ^1^, ^23^, ^24^, ^25^ ^]^ In this study, we utilized a biodegradable CNC composed of Mo/PCL, which exhibited a conductivity of ≈7.4 ± 1.9 S cm^−1^ (20 vol.% of Mo). The elongation at break values of previously investigated CNCs composed of a conducting polymer (PPy/SF) and graphene materials (GO/PCL) are ≈29%^[^ ^22^ ^]^ and 42.3%,^[^ ^23^ ^]^ respectively. As a result, when using 20 vol.% of Mo, the elongation break value falls within the values reported in the previous studies. The live/dead assay was performed to show cell viability under the same culture condition with 20 vol.% of Mo/PCL and control (cultured on coverslip glass), resulting in similar cell counting. Based on the cell viability result, the sciatic nerve conduit is safe for the cells in vitro (Figure S7, Supporting Information). Figure 2d shows the durability test results of the Mo/PCL composites (20 vol.% of Mo MPs) during the cyclic bending at various bending radii (1, 5, and 10 mm) considering various peripheral nerve dimensions. Even at the rat's nerve radius of 1 mm, the conductivity was maintained up to 10 000 cycles with negligible deviations, and it was judged to be suitable as a CNC.

In addition, the variations in electrical conductivity upon the immersion of Mo/PCL composites (20 vol.% of Mo MPs) into various biomimetic solutions, including Hank's balanced salt solution (HBSS), phosphate‐buffered saline (PBS) with 1× and 10× concentrations, and fetal bovine serum (FBS), for 10 days at 37 °C, were measured (Figure 2e). The conductivity increased rapidly on the first day followed by a gradual decrease upon the immersion for 10 days for all solutions. A possible reason for the increase in conductivity may be the removal of native oxides at the early stage of dissolution as previously reported for a Mo/PBAT paste.^[^ ^52^ ^]^ In the literature, the degradation of Mo was studied in vitro and in vivo.^[^ ^30^, ^53^ ^]^ In a simulated physiological medium, pure Mo undergoes progressive weight loss and consistent degradation with surface corrosion that occurs in a linear fashion. The overall dissolution reaction for Mo is:

(2) $$\mathrm{Mo}+4H_{2}O\to {\mathrm{MoO}}_{4}^{2-}+8H^{+}+6e^{-}$$

At a pH of 7.4, MoO_4_^2−^ ions, which can be metabolized, are the main species found in aqueous media. The in vivo dissolution rate of Mo is 12, 33, and 36 µg cm^−2^d after 3, 6, and 12 months for the samples implanted in the aortic vessel wall.^[^ ^30^ ^]^ The degradation behavior pattern is found to be comparable for in vitro and in vivo experiments.^[^ ^30^, ^53^ ^]^ Consequently, Mo/PCL represents the only CNC that is entirely biodegradable in the body without causing problems after degradation. Figure S5 (Supporting Information) shows conductivity changes up to 1 month under lipase with 37 °C PBS 1X. The conductivity initially increased and then decreased to 5.4 S cm^−1^ after 30 days. However, as it remained conductive afterward, it demonstrated suitability to provide sufficient electrical stimulation to the nerve. Figure 2f shows a change in uniaxial tensile stress–strain after immersion into 1× PBS solution for 21 days. As the immersion time in PBS increases, elongation and tensile strength decrease because of water penetration at the interface between the particle and the polymer, accelerating the hydrolysis rate.^[^ ^49^ ^]^ Based on the SEM images investigating the surface morphology of Mo/PCL composites upon their immersion into 1× PBS at 37 °C for 0, 7, 14, and 21 days, no significant changes in surface morphology were observed because PCL is a relatively slow‐dissolving material (Figure 2g).

The addition of a bioresorbable lubricant such as tetraglycol (TG) to Mo/PCL composites improves the conductivity and elongation due to more uniform dispersion of Mo MPs (Figure 2h,i,j). Figure 2h shows the conductivity of Mo/PCL composites with 20 vol.% of Mo MPs depending on the volume fraction of the added TG. When 3 vol.% of TG was added, the conductivity increases to ≈13.3 ± 4.06 S cm^−1^, which is even higher than that of the composites with the Mo MP volume fraction of 30%. We assume that the addition of TG improved the distribution of Mo MPs in the PCL matrix resulting in increased conductivity.^[^ ^52^ ^]^ However, when 4.5 vol.% of TG was added, the conductivity decreases slightly, which might be due to the interference of TG with the conductive path of Mo MPs in the PCL matrix. Figure S6 (Supporting Information) presents the time‐dependent electrical conductivity of the Mo/PCL/TG composites (20 vol.% of Mo MPs and 3 vol.% of TG) upon immersion into the 1× PBS solution at 37 °C. The electrical conductivity rapidly increases on the first day and then gradually decreases thereafter, which is in line with the electrical conductivity results without the addition of TG as previously shown in Figure 2e.

Further, the mechanical properties of Mo/PCL/TG composites with various volume fractions of TG were investigated using the uniaxial tensile test. As shown in Figure 2i, the elongation is improved by more than 5 times compared to the Mo/PCL composites without TG. Figure 2j presents elongation results according to the volume fraction of TG. The elongation is increased to 256% when 3 vol.% of TG was added. Thus, the addition of an appropriate lubricant amount to the conductive composites can increase its elongation by absorbing the stress concentrated at the interface between the polymer matrix and the metal particles.^[^ ^54^, ^55^ ^]^

### 2.3 In Vivo Therapeutic Treatment of Long Nerve Defect of Peripheral Nerve

**Figure 3:**
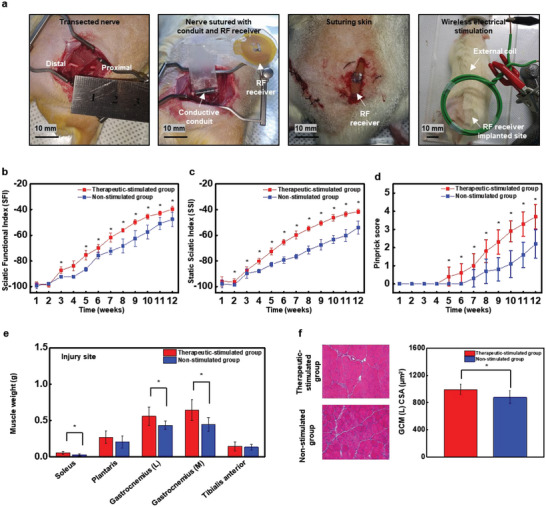
Surgical procedure, motor and sensory functional behavior test result, and histological results of muscle of therapeutic‐stimulated model and non‐stimulated group. a) Surgical procedure for implanting integrated systems. From left to right: the skin is incised and nerve is transected with 10 mm gap; transected nerve is sutured with biodegradable conductive nerve conduit; RF receiver is subcutaneously implanted and skin is sutured; electrical stimulation is activated with external coil. b) Sciatic functional index score for each week after transplantation for each group (*n* = 8). c) Sciatic static index score for each week after transplantation for each group (*n* = 8). d) Weekly pain level testing after transplantation, pin prick test (*n* = 8). Bars represent standard deviation of the mean.*p < 0.05 compared with each group by the Mann–Whitney U test. e) Wet weight measurement for each muscle type (*n* = 8). Bars represent standard deviation of the mean. *p < 0.05 compared with each group by the Mann–Whitney U test. f) Representative imaged of H&E staining result of lateral gastrocnemius muscle 12 weeks after biodegradable conductive nerve conduit (left). Cross section area measurement per unit muscle fiber (right) (*n* = 8).

**Figure** **3** shows in vivo procedures and the efficacy of therapeutic stimulation to damaged sciatic nerves. Following sciatic nerve transection in a nerve defect ≈10 mm, electroceutical (CNC + wireless electrical stimulator) was implanted between proximal and distal stumps and sutured. The RF receiver was placed just below skin layer, and skin layer was sutured owing to the absence of abnormalities on the skin or muscle reported by a previous study that used similar‐sized wireless electrical stimulator and employed hematoxylin and eosin (H&E) staining after inserting the wireless electrical stimulating system under the skin.^[^ ^29^, ^56^, ^57^ ^]^ Further, since the system is inserted under the skin and disconnected from external equipment, there is low risk of additional infection. Furthermore, the absence of any inflammatory response in the implanted electroceutical platform after 12 weeks indicates its biocompatibility (Figure S8, Supporting Information). Electrical stimulation conditions with monophasic pulse for nerve regeneration involve parameters such as pulse duration, stimulation pulse frequency, and the number of stimulation days. Despite the lack of optimization of the exact conditions for nerve regeneration, 100 µs pulse duration and 20 Hz frequency are mainly used, and it is observed that stimulation within an hour a day is more effective than continuous stimulation.^[^ ^39^, ^44^ ^]^ In this study, to regenerate the long nerve gap, electrical stimulation was transmitted to the RF receiver‐implanted site with monophasic therapeutic stimulation (100 µs pulse duration, 20 Hz frequency) three times a day for 1 h from the day of the surgery, as shown in Figure 3a. The experimental group was divided into two groups: one group was therapeutic‐stimulated with a CNC, Mo/PCL, and the other group did not receive stimulation with CNC (Figure S9, Supporting Information). Comparison between the experimental groups can be sufficiently compared through the sciatic functional index (SFI). Thus, in this experiment, we attempted to quantify the effect of electrical stimulation after inserting a CNC. In addition, it was confirmed whether electrical stimulation was transmitted from the proximal nerve stump to the distal nerve stump according to the presence or absence of CNCs (Figure S10a,b, Supporting Information). Electrical stimulation was well transmitted to the distal stump when CNC was present (Figure S10c, Supporting Information) but was not transmitted in the absence of CNC (Figure S10d, Supporting Information). This shows that the high‐conductivity CNC increases the electrical stimulation efficiency.

The SFI and static sciatic index (SSI) were evaluated to detect motor function, and to check sensory function, the pinprick test was performed every week until 12 weeks (Figure 3b–d). The SFI, quantified by analyzing rat walking tracks, is known to be a reliable method to assess functional recovery after sciatic nerve injury and repair.^[^ ^58^ ^]^ The SFI value ranges from −100 to 0, with a normal SFI falling in the range of 0 ± 10 and −100 indicating total loss of nerve function. The motor recovery expressed by SFI was enhanced more in the therapeutic‐stimulated group than in the non‐stimulated group at 3 weeks and 5–12 weeks after implantation (Figure 3b). The SFI values in Mo/PCL+therapeutic‐stimulated group and the non‐stimulated Mo/PCL group were −39.58 ± 2.36 and −47.56 ± 5.48, respectively, at 12 weeks after implantation. When comparing cases where only the CNC was used without electrical stimulation, the SFI was −47.5 ± 2.3 at 3 months after inserting the PPy/PDLLA CNC into a 10 mm gap.^[^ ^16^ ^]^ In this study, the SFI of autograft was −43.6 ± 2.5 after 3 months. When comparing the SFI values ​​between experiments using only the CNC, similar values ​​were obtained, and it was confirmed that the SFI value of the Mo/PCL+therapeutic‐stimulated group was higher than that of autograft. Compared to the study involving CNC with electrical stimulation, the SFI after 3 months with the PPy/SF CNC was −48.23 ± 5.15.^[^ ^22^ ^]^ Therefore, it was confirmed that the SFI of the Mo/PCL+therapeutic‐stimulated group showed a relatively higher value, which indicated that electrical stimulation with the Mo/PCL conduit is effective for functional recovery. Moreover, the SSI of the Mo/PCL+therapeutic‐stimulated group was higher than that of the non‐stimulated group from 2 to 12 weeks after implantation (Figure 3c). The pinprick score is 5 for normal and 0 for a total loss of nerve sensory function. The pinprick test showed better recovery in the therapeutic‐stimulated group than in the non‐stimulated group from 5 to 12 weeks after implantation (Figure 3d). Therefore, electrical stimulation proved effective at promoting axonal regeneration and regaining concomitant sensory and motor functions after peripheral nerve damage in animal models.

Twelve weeks after implantation, all subjects were sacrificed, and damaged sciatic nerves and denervated gastrocnemius muscles were obtained. The muscle weight of soleus, lateral, and medial gastrocnemius muscles and tibialis anterior muscles in the therapeutic stimulation group was heavier than those in the non‐stimulation group (Figure 3e). The muscle weight of the intact site is shown in Figure S11 (Supporting Information). Figure 3f shows that the cross‐sectional area (CSA) of reinnervated lateral gastrocnemius muscles in the therapeutic‐stimulated group (990.80 ± 76.33 µm^2^) was bigger than those in the non‐stimulated group (878.33 ± 96.25 µm^2^).

**Figure 4:**
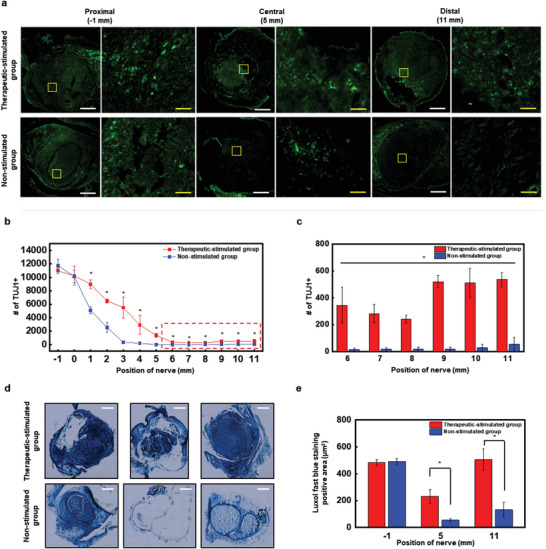
Histological results of therapeutic‐stimulated model and non‐stimulated group. a) Representative images of immunohistochemical staining result cross sectioned 12 weeks after biodegradable conductive nerve conduit (*n* = 8). The left images in proximal, center, and distal parts are low‐magnification images, while the right images are high‐magnification images of yellow boxes in the left images. White scale bar = 500 µm; yellow scale bar = 50 µm. b) The number of TUJ1‐positive axons per 1 mm section from the proximal section was counted (*n* = 8). c) Count of the TUJ1‐positive axons in the 6–11 mm distal section that passed through the injury site in units of 1 mm (n = 8). d) Luxol Fast Blue staining results for each area (−1 mm, +5 mm, +11 mm) (*n* = 8). e) Measurements of the area of positively myelinated axons stained with Luxol Fast Blue (*n* = 8). Bars represent standard deviation of the mean. *p < 0.05 compared with each group by the Mann–Whitney U test.

**Figure** **4**a shows representative images of immunohistochemical staining of sciatic nerves of the therapeutic‐stimulated and non‐stimulated groups in the proximal part (1 mm proximal to proximal stump), center of the nerve conduit, and distal part (1 mm distal to distal stump). A detailed transverse section of the sciatic nerve is shown in Figure S12 (Supporting Information). Statistically significant differences in the number of regenerated TUJ1‐postive axons began to appear from the 1 mm section between the therapeutic‐stimulated group (8980 ± 661) and non‐stimulated group (5090 ± 497) (Figure 4b). At the start of the nerve transection section, the difference was ≈10 times (therapeutic‐stimulated group: 536 ± 53 versus non‐stimulated group: 57 ± 50) in the 11 mm section beyond the damaged area (Figure 4c). One important aspect of neuro‐regeneration and functional recovery is the regeneration of axons, but it is also crucial to confirm the myelination of regenerated axons. To achieve this, we conducted Luxol Fast Blue staining, which stains myelinated axons blue and enables visualization of only functional axons (Figure 4d). As shown in Figure 4e, staining was performed at three locations: 1) 1 mm proximal to the injury site, 2) 5 mm distal to the injury site where the CNC is present, and 3) 11 mm distal to the injury site where the CNC ends. At the −1 mm location, there was no significant difference between the groups (therapeutic‐stimulated group: 483.64 ± 20.14 µm^2^ versus non‐stimulated group: 491.73 ± 21.64 µm^2^). However, at +5 mm (therapeutic‐stimulated group: 231.62 ± 51.22 µm^2^ versus non‐stimulated group: 54.59 ± 14.29 µm^2^) and +11 mm (therapeutic‐stimulated group: 504.22 ± 80.55 µm^2^ versus non‐stimulated group: 132.61 ± 53.46 µm^2^), statistically significant differences were observed between the groups. In this study, we did not conduct molecular analysis, however, possible meles.^[^ ^32^, ^59^, ^60^, ^61^ ^]^ Electrical stimulation has been shown to enhance neurotrophic factor expression, such as brain‐derived neurotrophic factor (BDNF) and its receptor trkB, which play a critical role in promoting axonal growth and survival.^[^ ^32^ ^]^ Additionally, electrical stimulation can activate intracellular signaling pathways that facilitate axonal sprouting and myelination.^[^ ^61^ ^]^ The application of electrical stimulation has also been demonstrated to accelerate axon outgrowth, guidance, and target reinnervation following nerve injury, potentially through the activation of growth‐associated genes.^[^ ^59^ ^]^ Furthermore, electrical stimulation increases Schwann cell activity, which is essential for nerve regeneration and remyelination.^[^ ^60^ ^]^ Collectively, these findings suggest that electrical stimulation promotes peripheral nerve regeneration through multiple mechanisms, including modulation of neurotrophic factor expression, activation of intracellular signaling pathways, and enhancement of axonal sprouting, myelination, and Schwann cell function.

### 2.4 Application Examples of Biodegradable Mo/PCL Composites

**Figure 5:**
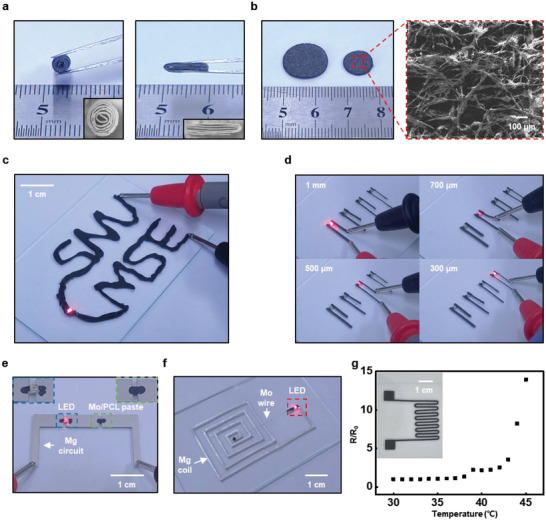
Application of biodegradable and conductive Mo/PCL composites in tissue engineering and printed electronics. a) Structure of a bone scaffold fabricated using the Mo/PCL composite. Inset shows an optical microscope image of the scaffold. b) Wound healing patch fabricated using the electrospun Mo/PCL composite with the corresponding SEM image. c) Conductive traces on a glass slide formed by direct printing of Mo/PCL dissolved in tetrahydrofuran (THF). d) Screen‐printed conductive traces of Mo/PCL composites with various line widths ranging from 300 µm to 1 mm. e) Mo/PCL conductive paste interconnecting Mg foils (50 µm thick). f) Wireless Mg inductive coil interconnected with the Mo/PCL paste can turn on/off an LED through wireless power transfer at a resonance frequency of 13 MHz. g) Temperature sensor produced by screen printing of the Mo/PCL composite.

**Figure** **5** demonstrates the extended applicability of biodegradable Mo/PCL composites with tunable geometry and microstructure in various tissue engineering fields and printed electronics. Figure 5a presents a cylinder‐structured scaffold formed by rolling and stacking the Mo/PCL composites with 20% of Mo MPs. The fabricated 3D bone scaffold can be applied to bone defects because it mimics the structure of bone tissue.^[^ ^62^ ^]^ Figure 5b shows a wound healing patch fabricated using an electrospun Mo/PCL composite with 20 vol.% Mo MPs with an average diameter of electrospun Mo/PCL being ≈4 µm and an SEM image revealing its microstructure. Compared to the Mo/PCL film fabricated by the drop‐casting method, the electrospun films consist of nanofibers, and their porosity can be adjusted by controlling the electrospinning conditions, providing scope for a broad range of modifications and applications in different tissue engineering fields.^[^ ^63^ ^]^

Biodegradable Mo/PCL composites can be applied in printing, as shown in Figure 5c–g. For the fabrication of electronic devices requiring high conductivity, the biodegradable Mo/PCL composite with 30 vol.% Mo MPs was used (Figure 5c–g). Figure 5c shows conductive traces directly written on a glass slide using Mo/PCL composite. An LED connected to the printed traces demonstrates stable operation as an interconnect electrode. In addition, Mo/PCL composite electrodes can be screen‐printed with various line widths between 300 µm and 1 mm with a thickness of ≈100 µm (Figure 5d). An LED was successfully connected with the same line width without heat treatment. Mo/PCL composites can also be used as conductive pastes (Figure 5e). An LED and an Mg circuit (50 µm thick foil) were interconnected using the Mo/PCL paste at room temperature as a demonstrative case. In another example shown in Figure 5f, an Mg inductive coil interconnected by Mo/PCL paste can wirelessly operate an LED. Printed Mo/PCL traces can also be applied to various sensors. In a resistive temperature sensor employing screen‐printed Mo/PCL composite, the resistance increases as the temperature increases, especially, there is a significant change above 40 °C (Figure 5g). Thermal expansion of the polymer binder may be responsible for this exponential increase in resistance.^[^ ^64^ ^]^

## 3 Conclusion

In this study, we fabricated an electroceutical platform to accelerate nerve regeneration in long nerve defects and improve functional recovery. The designed electroceutical consisted of a fully biodegradable CNC fabricated using metallic Mo MPs and PCL and a wireless electrical stimulator. A Mo/PCL composites can offer CNC that is fully biodegradable and has controllable conductivity. Wireless electrical stimulation was achieved through a fully implantable electrical stimulator to minimize side effects, such as inflammation during the stimulation for several days. The electrical stimulation applied to the proximal stump transmits signals through the CNC, creating an environment that guides the nerve to grow correctly and accelerates nerve regeneration. In an in vivo experiment, our biodegradable CNC, which bridged a 10 mm long rat's sciatic nerve gap, was stimulated for 1 h a day for a total of 3 days. The nerve regeneration and reinnervation were successfully achieved in the therapeutic stimulated model based on the nerve and muscle histology results. In addition, the motor and sensory function recovery was up to 7 weeks faster with electrical stimulation than without stimulation. Based on our results, the biodegradable CNC using biodegradable metal particle/polymer composites and wireless electrical stimulation accelerated nerve regeneration and improved function recovery. Further efforts devoted to a detailed understanding of the optimal stimulation conditions and chemical composition of the biodegradable conduit are needed to improve the success rate of nerve regeneration. Consequently, this study reports the only CNC that is entirely biodegradable in the body without causing problems after degradation. Also, current non‐degradable RF receiver can be substituted with a biocompatible and biodegradable RF receiver, which has been achieved in previous studies using Mg coil, Si nanomembrane diodes, and Mg/SiO_2_/Mg capacitors.^[^ ^29^, ^57^, ^65^ ^]^

## 4 Experimental Section

### Preparation of Mo/PCL Composites

PCL (*M*_n_ = 80000, Sigma–Aldrich, Germany) and tetrahydrofuran (THF, Junsei Chemical Co., Ltd., Japan) were mixed at a weight‐to‐volume (w/v) ratio of 1:10 until a viscous solution was obtained. Subsequently, 20 vol.% of Mo MPs (≈1.33 ± 0.38 µm, US Research Nanomaterials, INC., USA) was added to a PCL solution under continuous stirring, followed by the drop‐casting on a glass substrate and drying at room temperature for over 6 h, resulting in thin films (≈100 µm) of Mo/PCL composites.

### Preparation of Mo/PCL Conduit and Electroceutical Platform

The outer tube was fabricated using a PCL solution. The PCL was cast on a glass substrate and dried at room temperature for over 6 h resulting in thin films (50–100 µm) of PCL. The inner tube was fabricated using a thin film of Mo/PCL. A stainless‐steel wire (stainless steel 316L wire Φ 50 µm, NewMet, UK) was attached to the PCL thin film using a very small amount of THF. The bilayer Mo/PCL conduit was achieved by rolling up the thin films on a tube with a diameter of 2 mm. The non‐degradable wireless stimulator fabricated by I.P.S. in Korea consisted of copper wire receiver coils and a polyimide substrate (diameter: 2 cm, thickness: 110 µm). The receiver coils had 17 turns of two layers with a 16 mm radius connected to a Schottky diode (40 V, 120 mA, Digi‐Key Electronics, USA) and a capacitor (10 pF, 5 V, elePARTS, Korea). The capacitor, diode, and stimulating electrode are soldered on the substrate containing the inductor coil and followed by encapsulation of all components with Ecoflex 0030. Finally, the integrated non‐degradable wireless stimulator was interconnected with the stainless‐steel wire of the conduit, resulting in an electroceutical platform for nerve regeneration.

### Characterization of Electrical Properties and Dissolution Behavior of Mo/PLC Composites

Electrical conductivities were calculated using the resistance and thickness of Mo/PCL composites. Impedance was measured using a potentiostat (CHI660E, CH Instruments, Inc., USA) in PBS within the frequency range between 0.1 Hz and 100 kHz. Dissolution tests were conducted at 37 °C in four different solutions: 1X and 10X PBS, HBSS, and FBS (all purchased from Gibco, USA). All solutions were changed every other day, and resistance was measured every day for up to 10 days. SEM (FEI, Quanta 250 FEG, USA) was used to investigate the morphology of the composite surface and the morphological change of the composite upon dissolution.

### Characterization of Mechanical Properties of Mo/PCL Composites

Mo/PCL and Mo/PCL/TG composite films were cut into a dumbbell shape (50 mm in overall length, 12 mm in shoulder width, 6 mm in narrow width, 0.1 mm in thickness, and 20 mm in gauge length) using a laser cutter (MD‐U1000C, Keyence, Japan). Uniaxial tensile tests were performed using a universal testing machine (Instron, USA) with an elongation speed of 20 mm min^−1^.

### Characterization of Fatigue Behavior Mo/PCL Composites

Fatigue bending tests of Mo/PCL composites (20 vol.% of Mo MPs) were conducted using a multi‐mode fatigue tester (CKMF‐12P, CKSI, Korea) at three different bending radii (R = 1, 5, and 10 mm) and the speed of 1 Hz per cycle. The electrical resistance during tests was measured for every 1000th cycle.

### Culturing and Seeding of PC12 Cells

The PC12 cells were purchased from the American Type Culture Collection (ATCC, Manassas, VA, USA) and cultured in complete RPMI 1640 medium (WELGENE, Gyeongsan, Republic of Korea) supplemented with 10% horse serum (HS, Corning life science, Glendale, AZ, USA), 5% fetal bovine serum (HS, Corning life science, Glendale, AZ, USA), and 1% penicillin/streptomycin (P/S, Sigma–Aldrich, St.Louis, MO, USA) at 37°C and 5% CO_2_ incubator. For each independent experiment, PC12 cells were seeded at a density of 50 000 cells per 1 mL on sciatic nerve conduit samples.

### Cell Viability

For live and dead cell assay, a fluorescent live/dead cell assay kit (L3224, Invitrogen Life Technologies) was used. The cells were incubated Calcein‐AM and EthD‐1 in Dulbecco's phosphate‐buffered saline (DPBS) for 30 min at room temperature (RT), and the samples were visualized under a confocal microscope (Carl Zeiss Inc., Oberkochen, Germany). Four images were taken from each well (*n* = 4), and the number of cells were counted using ImageJ software (1.37 v, National Institutes of Health, Bethesda, MD, USA).

### Therapeutic Stimulation

Therapeutic monophasic electrical signal (duration: 100 µs, frequency: 20 Hz) was generated using a function generator (AFG31000 Series, Tektronix, USA) and amplified using an amplifier (210L, Electronics & Innovation, USA). The resulting output signal was delivered to an external coil consisting of three turns of wire 60 mm in diameter. The RF receiver coil received radio frequency power from the external coil. Therapeutic electrical stimulation was applied to the injured site of the rat's sciatic nerve in anesthesia states for 1 h (100 µs, 20 Hz) three times every day after the implantation of the nerve conduit into the transected sciatic nerve. During each electrical stimulation, the rat is maintained in an anesthetized state.

### In Vivo Surgical Procedure

SD rats (14 weeks, male, 400–420 g) were used for in vivo studies. All animal care and surgical procedures complied with the regulations of the Dankook University Animal Ethics Committee (approval number: DKU‐20‐035). All experimental animals were managed in satisfactory conditions with adequate water and fed in individual cages at a specific pathogen‐free facility. The facility was maintained at a temperature of 23–25 °C and a humidity of 45–50%. In model production, anesthesia was administered with isoflurane, and the skin and subcutaneous layers of the surgical site were incised with scissors, and the muscle layer was retracted to expose the sciatic nerve. Under a surgical microscope, the sciatic nerve was completely transected using micro‐scissors, and a 10 mm long nerve gap was made. The conductive Mo/PCL conduit was implanted between the nerve gap, and the proximal and distal stumps were sutured using 10‐0 Nylon. After implantation, the surrounding muscles and skin were sutured in the reverse order of opening. A total of 16 sciatic nerve transection models were made. In the experimental group (*n* = 8), the CNC (Mo/PCL) was inserted and therapeutic‐electrical stimulation was performed. In the control group (*n* = 8), only the CNC (Mo/PCL) was implanted. The animal models were observed 12 weeks after surgery and sacrificed.

### Motor Function Test, Sensory Test

Both the control and experimental groups underwent behavioral tests through weekly gait analysis up to 12 weeks after conductive Mo/PCL conduit and electroceutical implantation. The sciatic functional index (SFI) and sciatic static index (SSI) score was obtained by measuring the length of the sole and affected part of the tendon, the width of the toes, and the width of the middle three toes as previously described.^[^ ^66^ ^]^ If the sciatic nerve damage is severe, the toe cannot be fully extended; thus, the score is a negative number. The closer the SFI and SSI are to 0, the better the recovery of the damaged nerve. The pin prick test was performed to determine the degree of sensory recovery.^[^ ^66^ ^]^ For this test, rats were placed on a stainless‐steel mesh and covered with an acrylic cover (30 × 30 × 30 cm) to restrict movement. Measurements were taken 30 min before acclimatization to the environment and after limiting movement for 20–30 min. The greatest stimulus intensity not producing a response was recorded. The degree of avoidance was measured by dividing the sole into 5 equal parts and stimulating each part with a pin to assess functional sensory re‐innervation.

### Muscle Histology

The gastrocnemius, soleus, and plantaris muscles were used for sampling. Muscle samples were measured for wet weight at the time of extraction. After extraction, muscle sample was embedded through the snap freezing method in which isopentane (Sigma–Aldrich, St. Louis, Missouri) was directly frozen in a bath of liquid nitrogen. Samples of the experimental group and the control group were cut into 10 µm thick cross‐sections using cryo‐cut (Leica Biosystems, Deer Park, Illinois). Muscle atrophy was morphologically observed by staining with hematoxylin‐eosin (Merck, Darmstadt, Germany), and the area of muscle fibers in each group was measured using Image J (National Institutes of Health).

### Nerve Histology

After surgery, the rats were allowed to walk freely without special fixation. Sciatic nerves from all groups were immunohistochemically stained 12 weeks following conduit transplantation. All rats were injected with saline under deep anesthesia, subjected to cardiac perfusion, and fixed with 4% paraformaldehyde. The damaged sciatic nerve was removed, fixed with 4% paraformaldehyde, and immersed in a 30% sucrose solution for 3 days. Tissue was formatted in M1 compound (Thermo Fisher Scientific) and cut in the sagittal or axial direction into 16 µm sections. Triton X‐100 (0.2%) was added to a 2% BSA/PBS solution and blocked with 10% normal serum Primary antibodies diluted in 2% bovine serum albumin (BSA)/phosphate‐buffered saline (PBS) solution (mouse TUJ1 monoclonal antibodies, 1:1000, Abcam; rabbit S‐100 polyclonal antibodies, 1:1000, Thermo Scientific, RP‐75723) were mixed at −22–4 °C. Secondary antibodies diluted in 2% BSA/PBS solution (FITC‐conjugated chloride anti‐rat IgG, 1:200; rhodamine‐conjugated chloride anti‐rabbit IgG, 1:200, Jackson ImmunoResearch Laboratories) were incubated at room temperature for 2 h. The section was treated with PBS with DAPI, mounted in Vectashield® (Vector Laboratories), and observed under a confocal microscope (Carl Zeiss). The total TUJ1 positive axis was calculated in sections using the semi‐automatic and previously described methods modified by using NIH ImageJ software (National Institutes of Health). Luxol Fast Blue staining was performed to confirm the signs of neuroregeneration, focusing on myelination. The samples used were frozen sections as mentioned previously and washed with PBS for ≈10 min to remove the M1 compound (Thermo Fisher Scientific), followed by an overnight reaction with Luxol Fast Blue reagent (Sigma–Aldrich) at 60 °C. Subsequently, de‐staining and color development were carried out using lithium carbonate (Sigma–Aldrich) solution and 70% ethanol. Finally, after washing with distilled water, the samples were mounted with a mounting solution using an alcohol series ranging from 70% to 100% ethanol and then examined under a microscope.

### Statistical Analysis

IBM SPSS Statistics 26 (IBM SPSS, Armonk, NY, USA) was used for statistical analysis, and all numerical data were reported as means ± standard deviations. The Shapiro–Wilk test was performed to examine the normal distribution of all quantified histological and functional data from each group, and parametric or nonparametric tests were chosen according to the result. In this experiment, the statistical significance of the cross‐sectional area axon count and electrophysiological evaluation was verified between the experimental and control groups using the Mann–Whitney U test. Repeated measures two‐way ANOVA (time and group) was used to compare the SFI, SSI, pin prick test, and thermal test during a 12 week experimental period between the control and experimental groups, and then the Bonferroni post hoc test was used to analyze data from each time point. Statistical significance was determined at *p* < 0.05

### Fabrication of Electrospun Scaffolds

Bone scaffolds and wound healing patches were fabricated using Mo/PCL composite dispersions employing an electrospinning machine (NanoNC, EP100, Korea). For this, Mo MPs were dispersed in the PCL and tetrahydrofuran solution as described earlier. For the electrospinning of the Mo/PCL dispersion, the applied voltage was 8 kV and a flow rate of 0.16 mL min^−1^ with a nozzle of 5 mm in diameter. The electrospun Mo/PCL was rolled and stacked to form a multilayered scaffold for bone repair and wound healing. SEM (FEI, Quanta 250 FEG, USA) was used to investigate the morphology of the electrospun Mo/PCL, and ImageJ software was used to measure the average diameter of electrospun Mo/PCL.

### Fabrication of Electronics using Mo/PCL Composites

A Mo/PCL composite dispersion (30% w/v) dissolved in PCL/THF (30% w/v) was directly printed on a glass slide using a syringe. For the fabrication of conductive traces, Mo/PCL composite dispersion (30% w/v) dissolved in PCL/THF (20% w/v) was spread on a glass slide using a blade. Hereby, Zn foil (thickness of 100 µm) served as a mask for Mo/PCL conductive lines with different widths (300 µm–1 mm, ≈100 µm in thickness). The Mg circuit and Mg coil were cut using a laser cutter and interconnected with an LED using a solder paste made of Mo/PCL composite (30% w/v) dissolved in PCL/THF (20% w/v).

## Conflict of Interest

The authors declare no conflict of interest.

## Supporting information

Supporting Information

## Data Availability

The data that support the findings of this study are available from the corresponding author upon reasonable request.
