# Supplementary material for: Electroceuticals for Regeneration of Long Nerve Gap Using Biodegradable Conductive Conduits and Implantable Wireless Stimulator
Source: Adv Sci (Weinh). 2023 Jun 20;10(24):2302632. doi: 10.1002/advs.202302632 (PMC10460856; doi:10.1002/advs.202302632)

ADVANCED SCIENCE
Open Access

Supporting Information

for *Adv. Sci.*, DOI 10.1002/advs.202302632

Electroceuticals for Regeneration of Long Nerve Gap Using Biodegradable Conductive Conduits and Implantable Wireless Stimulator

Jio Kim, Jooik Jeon, Ju-Yong Lee, Badamgarav Khoroldulam, Sung-Geun Choi, Jae-Young Bae, Jung Keun Hyun and Seung-Kyun Kang**

Supporting Information

Electroceuticals for Regeneration of Long Nerve Gap using Biodegradable Conductive Conduits and Implantable Wireless Stimulator

Jio Kim†, Jooik Jeon†, Ju-Yong Lee, Badamgarav Khoroldulam, Sung-Geun Choi, Jae-Young Bae, Jung Keun Hyun*, and Seung-Kyun Kang*

Corresponding authors. Email: kskg7227@snu.ac.kr (S. -K. Kang); rhhyun@dankook.ac.kr (J. K. Hyun)

Figure S1. The SEM image of Mo MPs.

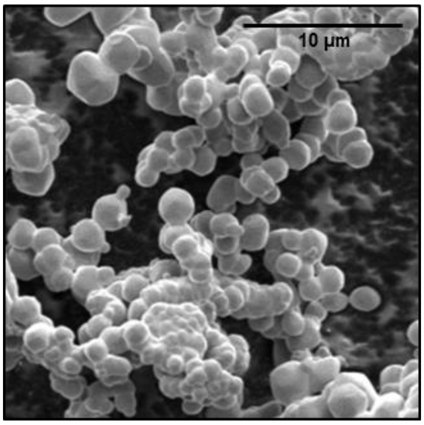

Figure S2. The structure of wireless electrical stimulator.

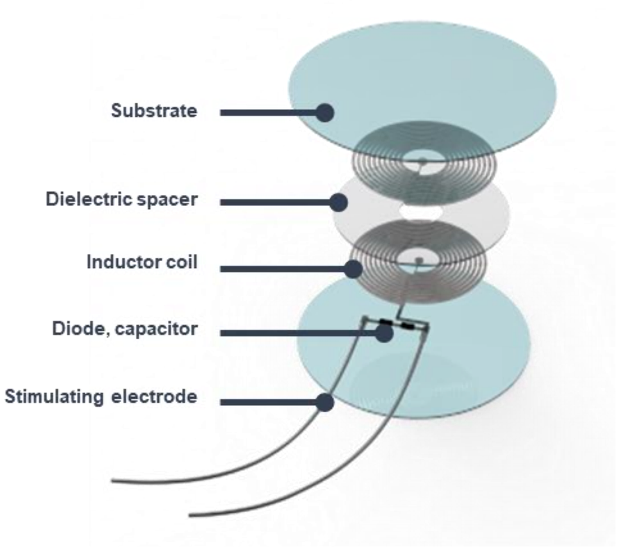

Figure S3. The rectifier circuit of the wireless electrical stimulator and external coil.

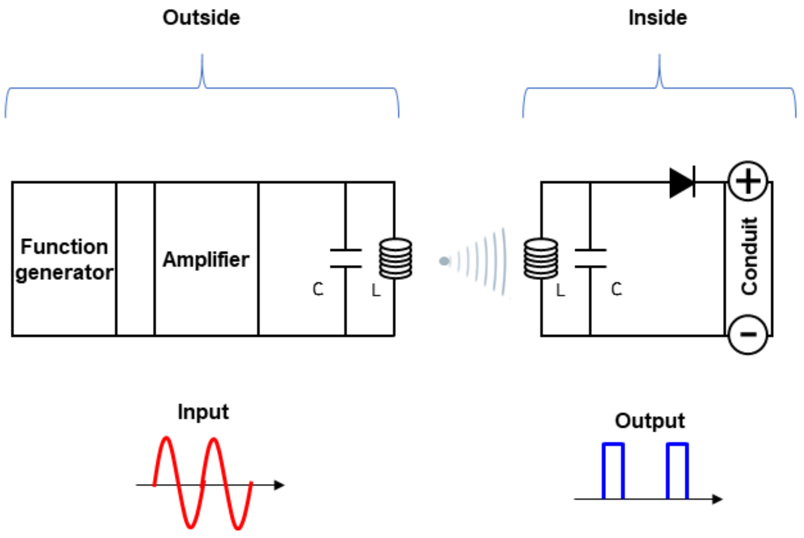

Figure S4. The SEM image of Mo/PCL composites with Mo vol.% 20 and 35.

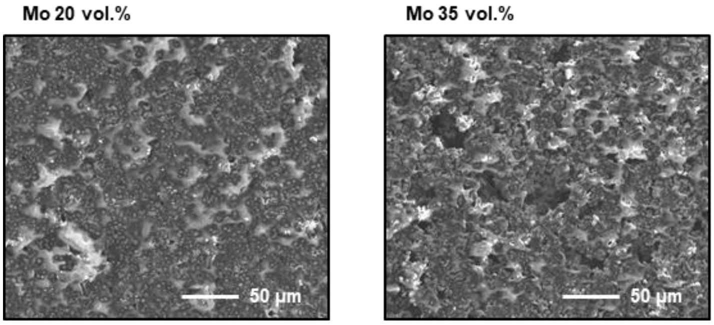

Figure S5. Time-dependent electrical conductivity of Mo/PCL composite (20 vol.% of Mo MPs) immersed into PBS 1X concentration with lipase at 37 °C.

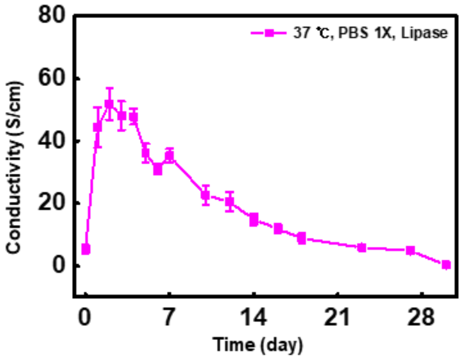

Figure S6. Change in conductivity with time of Mo/PCL/TG composites.

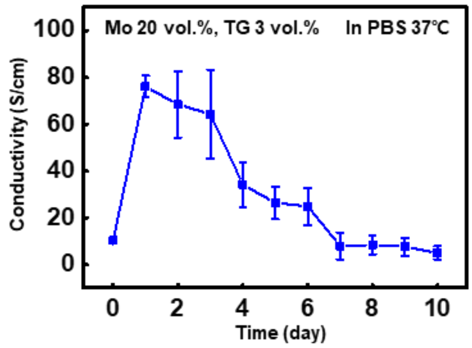

Figure S7. Live/dead test on Mo/PCL. a) Fluorescence microscopy images of live/dead assay. Scale bar = 200 µm. b) Cell viability obtained from live/dead assay. Data shown are the mean ± SD, n =3.

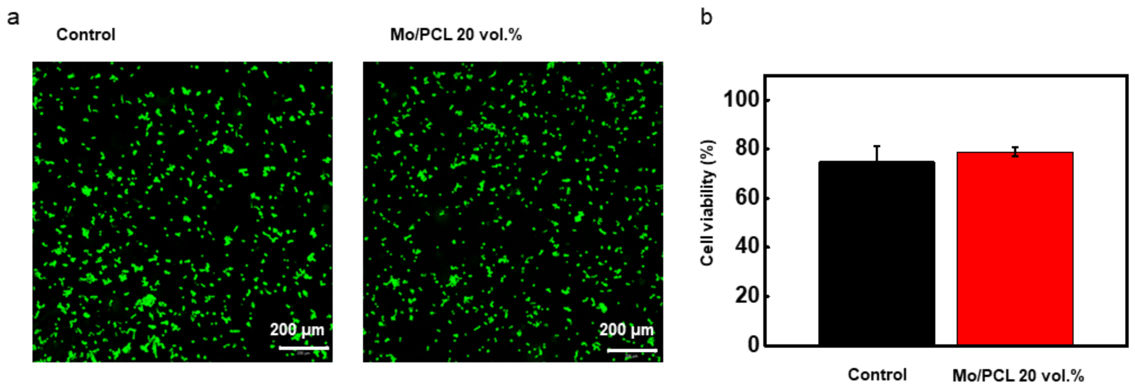

Figure S8. Photographs of subcutaneous view at 12 weeks after implantation.

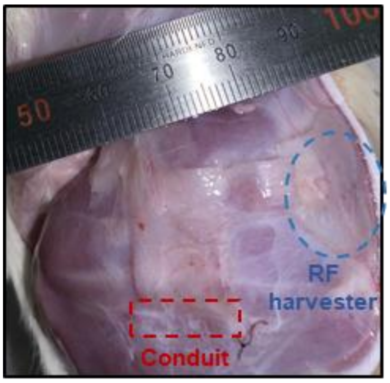

Figure S9. In vivo demonstration model image a) therapeutic-stimulated model with Mo/PCL CNC (n=8), b) non-stimulated model using Mo/PCL CNC (n=8).

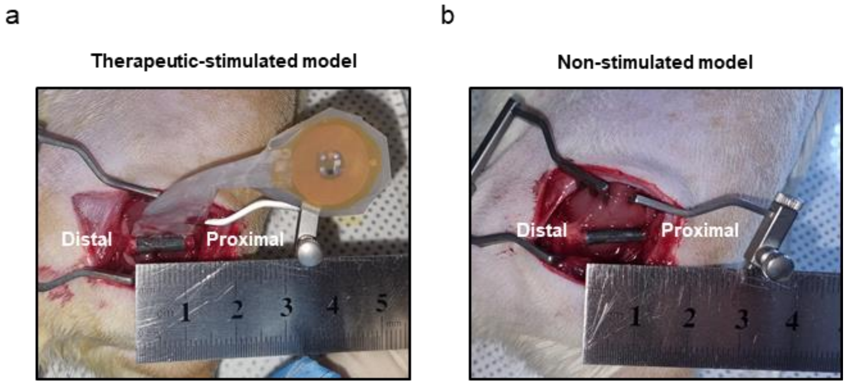

Figure S10. Verifying the signal transmitted through the implanted conductive nerve conduit. a) The experimental set up b) In vivo experiment details c) Results of signal transmitted through the Mo/PCL d) Results of signal transmitted through the PCL (non-conductive material).

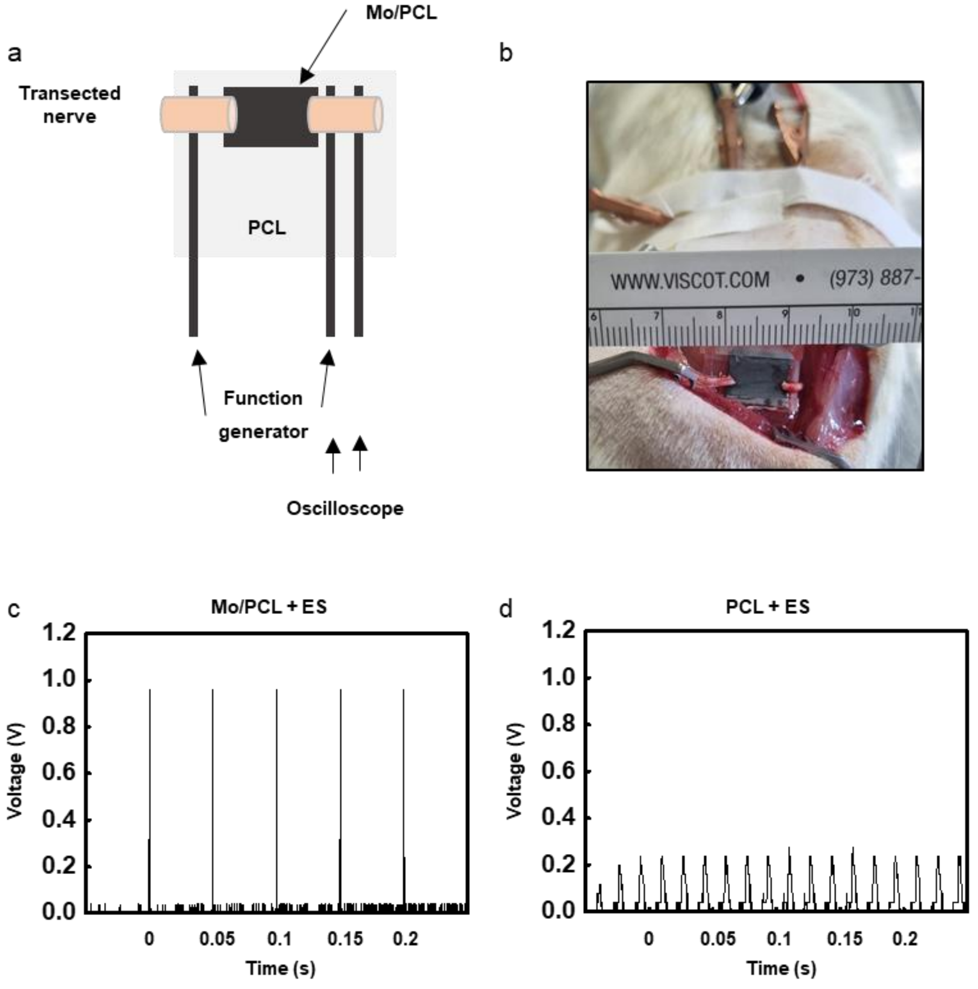

Figure S11. Muscle weight of intact site for each muscle type.

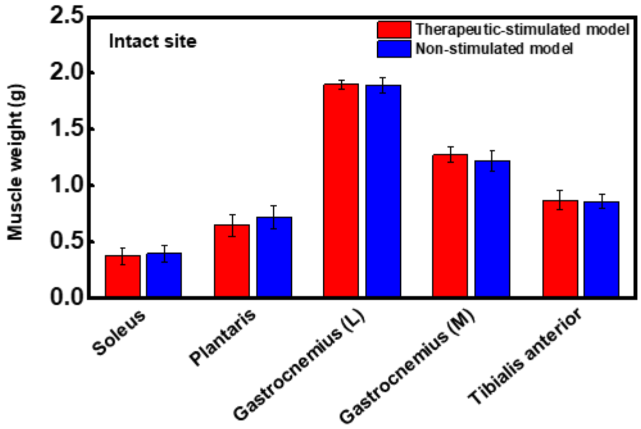

Figure S12. The transection points of sciatic nerve with conductive nerve conduit.

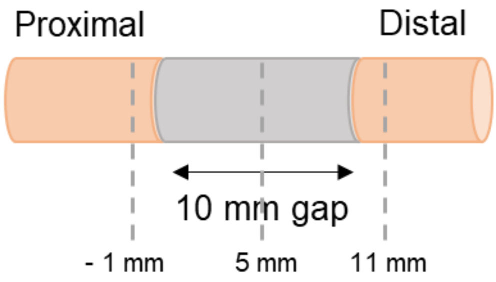

Supplement: Supplementary file 1 — Supporting Information [file ADVS-10-2302632-s001.pdf]
